# Supplementary material for: Development of a novel index to characterise arterial dynamics using ultrasound imaging
Source: PLoS One. 2021 Mar 4;16(3):e0248043. doi: 10.1371/journal.pone.0248043 (PMC7932503; doi:10.1371/journal.pone.0248043)
Supplement: S1 Table — Participant baseline demographics were collected at the time of enrollment to the study. Characteristics that follow a Gaussian/Normal Distribution are presented using mean ± SD, and cohort differences are compared using a student t-test. For variables that don’t follow a Gaussian distribution, median and inter-quartile range (IQR) are presented and cohort differences are compared using a Mann-Whitney test. BMI: body mass index. CAD: coronary artery disease, PAD: peripheral arterial occlusive disease, ACEi: angiotensin converting enzyme inhibitor; ARB: angiotensin receptor blocker. (PDF) [file pone.0248043.s001.pdf]

	AAA patients with measurable BARI (n = 114)	p Value
**Male (%)**	87.5%	<0.01**
**Age at Consent (Median/IQR)**	76 (69 – 81)	0.89
**BMI (Median/IQR)**	27 (24 – 30)	0.84
**Smoking History (%)**	84.8%	0.04*
**CAD History (%)**	40.2%	0.27
**PAD History (%)**	21.4%	0.25
**Cerebral Arterial Disease History (%)**	14.3%	0.89
**Hypertension (%)**	63.4%	0.04*
Mean arterial pressure (mmHg ±SD)	95 (20)	0.86
**Hypercholesterolemia (%)**	57.1%	0.64
**Diabetes (%)**	17.9	0.04*
HbA1C (Median/IQR)	5.7 (5.5 – 6.1)	0.08
**ACEI/ARB (%)**	64.2%	0.04*
**Anti-coagulants (%)**	11.5%	0.62
**Anti-platelets (%)**	58.7%	0.06
**Beta-Blockers (%)**	40.7%	0.08
**Calcium channel blockers (%)**	24.7%	0.60
**Diuretics (%)**	23.9%	0.93
**Gastro-restraint (%)**	36.3%	0.58
**Steroids (%)**	10.6%	0.60
**Statins (%)**	72.6%	0.74
**AAA Diameter, mm (Median/IQR)**	45 (37 - 52)	

Supplementary Table 1: **Summary of participant demographics and their association with BARI.** Participant baseline demographics were collected at the time of enrollment to the study. Characteristics that follow a Gaussian/Normal Distribution are presented using mean ± SD, and cohort differences are compared using a student t-test. For variables that don't follow a Gaussian distribution, median and inter-quartile range (IQR) are presented and cohort differences are compared using a Mann-Whitney test. BMI: body mass index. CAD: coronary artery disease, PAD: peripheral arterial occlusive disease, ACEi: angiotensin converting enzyme inhibitor; ARB: angiotensin receptor blocker.
