# Supplementary material for: Development of a novel index to characterise arterial dynamics using ultrasound imaging
Source: PLoS One. 2021 Mar 4;16(3):e0248043. doi: 10.1371/journal.pone.0248043 (PMC7932503; doi:10.1371/journal.pone.0248043)
Supplement: Attachment — Submitted filename: Response to Reviewers PLOS One.docx [file pone.0248043.s003.docx]

**Response to Academic Editor and Reviewers’ comments**

**Thank you very much for submitting this interesting Article to PLOS ONE.**

**Some major comments / issues were raised by the Reviewers, who are both world-class experts in diagnosis and management of aneurysmal disease. We would be most grateful if you could please address all points raised, but most importantly explain why this specific artery was chosen (radial). Please address the comment(s) raised about other arterial segments such as the popliteal artery (especially since this is the most common site of aneurysmal disease).**

**Response**:

We are grateful for the appreciation of the importance of the topic and feedback from all who reviewed the manuscript. We have specifically addressed these points in turn below and hope that the revised manuscript adequately addresses the raised points and is of suitable quality for publication in PLOS One.

**1. Why choose the brachial artery? Ease of access is obvious but there is some data to suggest that AAA is a systemic dilating disease and the popliteal artery (with its association with AAA) was chosen.**

**Response:**

We thank the Reviewer for raising this question, which certainly requires further clarification. This current project is an extension of our prior work, where we reported a significant correlation between flow mediated dilatation (FMD) of brachial artery and the future AAA growth of an individual. *As part of the research assessment, we acquired the video recording of the brachial artery at the same time as we measured flow mediated dilatation (FMD) of the brachial artery.* FMD is an induced response, driven largely by the endothelial nitric oxide bioavailability. This led us to hypothesise that intrinsic physical viscoelastic characteristics of the brachial artery will correlate with AAA growth. We devised brachial artery relaxation index (BARI) in the methods described in the manuscript.

Although it should be feasible to measure the viscoelastic properties in other major arteries (such as at the popliteal artery), we did not perform this during this study. As the Reviewer mentioned, ease of access is an obvious consideration. In this regard, common femoral artery is another potential site that can be accessed with ease. We had simply chosen the brachial artery for pragmatic reasons, as discussed above.

**2. Have you allowed for confounding factors? Many of these patients will also have other risk factors (high BP, smoking etc) - does this affect your results? Do drugs affect the relaxation index? The patients were all studied during a clinical visit that can be stressful? Did you make allowances for this?**

**Response:**

We thank the Reviewer for raising these important points. As we did not observe a correlation between BARI and future AAA growth (nor any difference in the other comparisons), we did not proceed to examine potential confounding effects of the other demographic variables. In response to the Reviewer’s comment and in preparation for this revision, we examined the association between BARI and the demographic variables as summarised in **Supplemental Table 1**.

It is interesting that BARI were influenced by some demographic variables (such as gender, history of smoking, prior diagnosis of hypertension or diabetes). We further observed a significant correlation between BARI and future 12-month aneurysm growth in female participants, however the number of females was small (**Figure below**) as one would anticipate from the usual case mix of AAA patients.

That BARI does not correlate with mean arterial pressure or HbA1C indicate that these patients have received appropriate medical therapy for their prior diagnosis of hypertension and diabetes. Among the recorded medications, the use of angiotensin converting enzyme (ACE) or angiotensin 2 receptor blocker (ARB) had significant effect on BARI. These two classes of medication have the similar effect of arterial relaxation. It is therefore not surprising that the use of either ACE or ARB can affect BARI, which is a measure of the viscoelastic properties of brachial artery.

The Reviewer aptly recognised that the clinical encounter can be a stressful experience for the study participants. To minimise the effect of this, we synchronised the research assessment with their regular NHS appointments for AAA surveillance scans. This minimised the inconvenience to the participants. Participant were required to fast for 6 hours before the study (except their regular medications) and to refrain from caffeinated beverages and cigarette smoking for 24 hours prior to their visits. To minimise stress, the study was performed in a dimly lit, quiet room, with the patient in the supine position separate to their clinical appointment. This was described in our original publication detailing the methods of FMD measurement.^1^ In response to the Reviewer’s comment, we have included the following in the revised manuscript:

Revised manuscript methods section (**Line 57 – 64**)

*The clinical encounter can be a stressful experience for the study participants. To minimise the effect of this, we synchronised the research assessment with their regular NHS appointments for AAA surveillance scans, during daytime hours. This minimised the inconvenience to the participants. Participants were required to fast for 6 hours before the study (except their regular medications) and to refrain from caffeinated beverages and cigarette smoking for 24 hours prior to their visits. To minimise stress, the study was performed in a dimly lit, quiet room, with the patient in the supine position separate to their clinical appointment. This was described in our original publication detailing the methods of FMD measurement.^1^*

Revised manuscript discussion section (**Line 220 – 228**)

*As we did not observe correlation between BARI and future AAA growth, we did not proceed to examine potential confounding effects of the other demographic variables. As a post hoc analysis, we examined the association between BARI and the demographic variables as summarised in Supplemental Table 1. It is interesting that BARI had significant correlation with some demographic variables (gender, history of smoking, prior diagnosis of hypertension or diabetes). We further observed a significant correlation between BARI and aneurysm growth in female, however the sample size of females was too small. That BARI does not correlate with mean arterial pressure or HbA1C indicate that these patients have received appropriate medical therapy for their prior diagnosis of hypertension and diabetes.*

**3. A null hypothesis and power calculation would help**

**Response:**

We thank the Reviewer for raising this important question. Based on our prior work, we hypothesised that intrinsic physical viscoelastic characteristics of the brachial artery will correlate with AAA growth. We first developed the method to extract a novel index to describe the viscoelastic properties of an artery (BARI), and subsequently examined its association with future AAA growth. Calculation for power required differs between studies seeking to find a predictive marker (such as in this study) from group comparisons. In our prior reports, prediction models of AAA growth were derived using AAA diameter and another biomarker as two input variables and no/slow growth (0% per year) as the primary outcome variable^2, 3^. For calculation of sample size requried for prediction models, a minimum of events (ie primary outcome variable) are required per input variables to evaluate its performance^4^. Therefore a minimum of 30 cases of no/slow growth are required to evaluate the prediction model in this context. In this recruited OxAAA cohort, 31 patients recorded no growth (0% growth) over the subsequent 12 month. It would have been reasonable to assess the performance of the prediction model (sensitivity, specificity etc) in this cohort, where 2 input variables are utilised (ie AAA diameter and BARI). However, as no correlation was observed between BARI and AAA growth, we did not proceed to assess the utility of BARI as a predictor of AAA growth.

Although it is possible that a significant correlation between BARI and AAA growth may be observed if the overall cohort size was much larger, our prior work has consistently shown that it is feasible to observe significant correlations in similar sized cohorts when a biological siginal is present.^1, 3, 5^

**--------------------------------------**

**Reviewer #2: Thank you for your well-written paper. I have the following comments:**

We thank the Reviewer for the very encouraging feedback and will address each of the comments accordingly.

**1. Page 3, Lines 22: The threshold of 55mm is not the only criterion. The expansion rate within one year as well as the shape of the aneurysm sac are playing an important role as well.**

**Response:**

We thank the Reviewer for raising this important point overlooked in the initial draft and have added and referenced a further sentence to recognise the alternative indications for intervention (Revised manuscript lines **24 - 26**).

“In addition to this, intervention is recommended for patients experiencing symptoms from their aneurysm, patients with saccular shaped aneurysms or those with aneurysms that expand quickly within one year.”

**2. Page 4, Line 39: Which is exactly the predictive power in percentage of the FMD in the brachial artery regarding the AAA identification?**

**Response:**

We wish to clarify that FMD of brachial artery (combined with AAA diameter) can be useful for predicting future growth, not for AAA identification as the Reviewer questioned here. In our prior publication^2^, we were able to predict an individual’s AAA diameter to within 2mm error in 85% and 71% of patients at 12 and 24 months.

**3. Is the BARI and index that it has been evaluated in other studies?**

**Response:**

We thank the Reviewer for raising this point. To our knowledge, BARI is a novel index discovered by our workflow and has thus not yet been evaluated in other studies. Indeed, the method pertaining to BARI analysis was subject to an international patent filing (WO/2020/030890).

**4. The different number of patients presented in the results is very confusing. It would be meaningful to provide a flow diagram with all measurements performed and the respective cohorts of patients.**

**Response:**

We thank the Reviewer for raising this and apologise for not making it easier to understand in the original text. To address this, we have created a figure (6) outlining the patient journey throughout the study as well as the data collected at each point in time. We attach the figure here for the Reviewer’s reference. We have revised the manuscript to update the numbers of the participants analysed in each of the comparison to reflect this. We hope this will make it easier to understand data collection and comparisons performed.

**5. Page 10, Line 193: Please provide literature about these preliminary data.**

**Response:**

We thank the Reviewer for raising this point. The preliminary data is from the pilot study, referenced on page 8 (**lines 154 – 167**). In brief, 6 patients with the fastest and slower growing aneurysms from the study cohort. fast growing and 3 with slow growing aneurysms. An initial analysis performed suggested that BARI could differentiate the two groups, hence the further analysis conducted throughout the OxAAA cohort (with these 6 patients included in the final results). The pilot study results are displayed in Figure 5.

**6. Page 10, Line 195: How time-consuming is to measure the BARI and how can we simplify it in order to investigate further cardiovascular pathologies**

**Response:**

We thank the Reviewer for considering the logistics of calculating BARI and how these could be improved to facilitate further use in the research setting. BARI can be quickly calculated using the detailed MATLAB algorithm within 5 minutes per set of images by a trained operator with appropriately powerful hardware. This process could be automated using machine learning if a suitably large dataset required analysis. However, acquisition of the required ultrasound images is more time consuming and not easy to expedite without compromising result quality, hence the rigorous protocol described in our previous open access publication.^1^

**7. Why did you select brachial artery and not radial artery? Is the size that makes the difference? Why not CFA or carotid artery or even better popliteal artery which is the second more frequent artery with aneurysmatic degeneration?**

**Response:**

We thank the Reviewer for this comment, which was also commented by Reviewer 1 (Comment 1). We have repeated the response here for Reviewer 2’s reference:

This current project is an extension of our prior work, where we reported a significant correlation between flow mediated dilatation (FMD) of brachial artery and the future AAA growth of an individual. *As part of the research assessment, we acquired the video recording of the brachial artery at the same time as we measured flow mediated dilatation (FMD) of the brachial artery.* FMD is an induced response, driven largely by the endothelial nitric oxide bioavailability. This led us to hypothesise that intrinsic physical viscoelastic characteristics of the brachial artery will correlate with AAA growth. We devised brachial artery relaxation index (BARI) in the methods described in the manuscript.

Although it should be feasible to measure the viscoelastic properties in other major arteries (such as at the popliteal artery), we did not perform this during this study. As the Reviewer mentioned, ease of access if an obvious consideration. In this regard, common femoral artery is another potential site that can be accessed with ease. We had simply chosen the brachial artery for pragmatic reasons, as discussed above.

**8. Do you think that further improvement of US device could provide more data regarding direct measurement of BARI in the abdominal aorta?**

**Response:**

The Reviewer raised a very astute comment. Indeed, we had thought of the same. In this same cohort of participants, we also recorded a video loop of the abdominal aorta (at the same time as we measured AAA diameter for surveillance). However, the resolution of these AAA video images were too low to calculate BARI. We are equally optimistic as the Reviewer that direct measurement of BARI at the aorta would be feasible in the future, with the improvement of ultrasound technology.

-----------------------

**Reference:**

1.	Lee R, Bellamkonda K, Jones A, et al. Flow Mediated Dilatation and Progression of Abdominal Aortic Aneurysms. *Eur J Vasc Endovasc Surg* 2017; 53(6):820-829.

2.	Lee R, Jarchi D, Perera R, et al. Applied Machine Learning for the Prediction of Growth of Abdominal Aortic Aneurysm in Humans. *EJVES Short Rep* 2018; 39:24-28.

3.	Lee R, Cassimjee I, Huang H, et al. Integrated Plasma and Tissue Proteomics Reveals Attractin Release by Intraluminal Thrombus of Abdominal Aortic Aneurysms and Improves Aneurysm Growth Prediction in Humans. *Annals of surgery* 2020.

4.	Harrell FE, Harrell FE. Regression Modeling Strategies: With Applications to Linear Models, Logistic Regression, and Survival Analysis (Springer Series in Statistics): Springer, 2006.

5.	Lee R, Fischer R, Charles PD, et al. A novel workflow combining plaque imaging, plaque and plasma proteomics identifies biomarkers of human coronary atherosclerotic plaque disruption. *Clin Proteomics* 2017; 14:22.
